# Bioinspired Cationic Antimicrobial Polymers

**DOI:** 10.1002/anie.202503738

**Published:** 2025-05-15

**Authors:** Heliya Javadi, Anne‐Catherine Lehnen, Matthias Hartlieb

**Affiliations:** ^1^ Institute of Chemistry University of Potsdam Karl‐Liebknecht‐Straße 24–25 14476 Potsdam Germany; ^2^ Fraunhofer Institute for Applied Polymer Research (IAP) Geiselbergstraße 69 14476 Potsdam Germany

**Keywords:** Antibacterial, Antimicrobial polymers, Antimicrobial resistance, Cationic polymers, Membrane interaction

## Abstract

Antibiotics are an essential tool of modern medicine, which is critically endangered by the spread of antimicrobial resistance (AMR). Without effective antibiotics, a number of medical advancements from the last century are in jeopardy, threatening our global public health and leading to a high death toll. To counteract this development, new therapeutic strategies, that are insusceptible to resistance development have to be established. Among them, antimicrobial polymers (AP)s are a promising class of materials. Their mode‐of‐action is highly unspecific as they kill bacteria by membrane permeabilization or precipitation of intracellular components. As such, it is unlikely for APs to be affected by AMR. This review highlights recent advances in AP design and understanding of structure–property relationships of these cationic macromolecules. One spotlight is on the polymeric architecture and how it influences AP bioactivity. A second highlight is stimuli‐responsive APs and their potential to increase AP selectivity. Moreover, synergistic effects, e.g., between polymer and antibiotics are reviewed. The last focus is on in vivo applications of APs, which could pave a way toward clinical applications.

## Graphical abstract

A review about membrane active cationic polymers with antimicrobial properties. A focus is laid on polymeric architecture, stimuli‐responsive antimicrobial polymers, as well as synergistic effects of polymers, and other compounds.

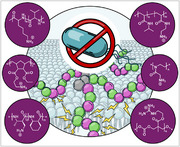

## 1 Introduction

The advent of antibiotics has drastically transformed medicine and health care. Without drugs like Penicillin, a plethora of standard medical treatments would simply be impossible. Chemotherapy of bacterial infections was pioneered by Paul Ehrlich, who developed Salvarsan as effective treatment for syphilis in 1910.^[^ ^1^ ^]^ But even after the discovery of Penicillin by Sir Alexander Fleming in the 1920′s,^[^ ^2^ ^]^ it was not until the 1940′s before antibiotic therapies became broadly available. Even then, the luring threat of antimicrobial resistance (AMR) was already obvious to some. Fleming stated, “The thoughtless person playing with penicillin treatment is morally responsible for the death of the man who succumbs to infection with the penicillin‐resistant organism” in an interview after receiving the Nobel prize for his research.

And indeed, among the problems endangering our global public health, AMR is a leading concern. A recent article summarizes the current burden to an estimated 4.71 million deaths associated with AMR, including 1.14 million deaths directly attributable to resistant bacteria in 2021 alone.^[^ ^3^ ^]^ This is connected to a dire forecast of approximately 10 million annual deaths in 2050. In particular the so called ESKAPE pathogens (*Enterococcus faecium*, *Staphylococcus aureus* (*S. aureus*), *Klebsiella pneumoniae (K. pneumoniae*), *Acinetobacter baumannii*, *Pseudomonas aeruginosa (P. aeruginosa)*, and other *Enterobacterales*) pose major problems as they are well adjusted to hospital settings.^[^ ^4^ ^]^

Misuse of antibiotics causes substantial pressure for pathogens to adapt, and has led to the rapid spread of resistances.^[^ ^5^ ^]^ The issue is aggravating, as pharmaceutical companies cease their investments in the development of new antibiotics, as the expected financial success of new compounds does not seem to justify the costly development and approval process.^[^ ^6^ ^]^ One main reason is that new antibiotics are immediately declared last resort treatments to prevent new resistances.

Hence, research efforts beyond the classic concept of antibiotics, leading to treatments that are not affected by AMR are highly desirable. Inspiration can be found in host‐defense peptides (HDP)s, which are used by the innate immune system of many organisms to combat pathogens.^[^ ^7^ ^]^ However, the direct utilization of peptides is associated with drawbacks, namely high costs, vulnerability to proteolytic enzymes, substantial toxicity, and their potential immunogenicity. Moreover, HDPs are not designed to be employed on a systemic level, but rather manage microorganisms on a very local scale, preventing an application along the lines of classical antibiotics. Thus, adapting the general concept of HDPs into a more modular platform is an auspicious strategy.

## 2 Antimicrobial Polymers

**Figure 1:**
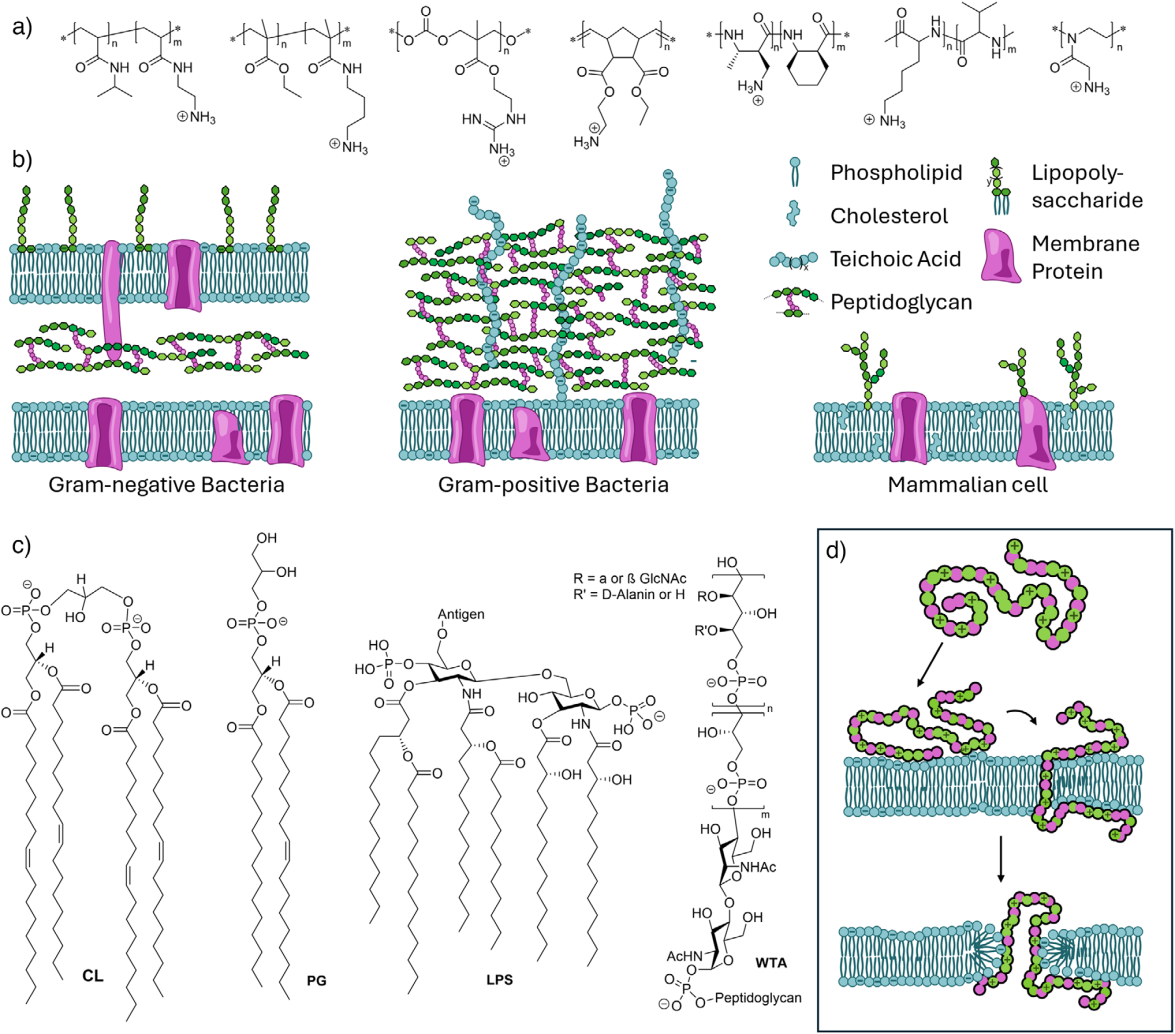
a) Selection of AP structures: (from left to right) poly(acrylamides),^[^ ^8^ ^]^ poly(methacrylates/acrylamides),^[^ ^15^ ^]^ poly(carbonates),^[^ ^16^ ^]^ poly(norbornenes),^[^ ^17^ ^]^ nylon,^[^ ^18^ ^]^ poly(aminoacids),^[^ ^19^ ^]^ and poly(oxazolines),^[^ ^11^ ^]^ b) schematic overview over different architectures/compositions of bacterial or mammalian cell envelope illustrating the surplus of negative charges in bacterial membranes; c) selection of anionic components of the bacterial cell envelope: PG (1‐palmitoyl‐2‐oleoyl‐sn‐glycero‐3‐phospho‐(1′‐rac‐glycerol)) – 55% in *S. aureus*, 20% in *Escherichia coli* (*E. coli*), CL (cardiolipin) ‐∼5% in *S. aureus* and *E. coli*, LPS (Lipopolysacharide) from *E. Coli*, WTA (wall teichoic acid) from S. aureus. d) Schematic illustration of membrane disruption interaction based on electrostatic interactions.

Antimicrobial polymers (AP)s were first reported in the early 2000′s and mimic the fundamental properties of HDPs, namely cationic charges, as well as hydrophobic subunits. With this combination APs can lyse the bacterial cell membrane, a mechanism that is sufficiently unspecific to avoid resistance development.^[^ ^8^, ^9^, ^10^, ^11^ ^]^ Still, abundance of negatively charged biomolecules in bacterial membranes enables selectivity over mammalian cells (Figure 1c). Initial efforts were focused on the implementation of facial amphiphilicity (separating cationic and hydrophobic moieties on opposite sides of a rather stiff macromolecule) as found in some HDPs.^[^ ^12^ ^]^ However, ultimately this was found to be nonessential as flexible polymers can adopt such a structure when in contact with the membrane,^[^ ^13^, ^14^ ^]^ which is also supported by computational modeling.^[^ ^15^ ^]^

In contrast to polymeric disinfectants, APs are designed to display a certain selectivity for bacterial cells. This ability is strongly coupled to the amphiphilic balance (i.e., the ratio between cationic charge and hydrophobicity).^[^ ^20^ ^]^ A surplus of either quality leads to unspecific toxicity against mammalian cells, and potent antimicrobial activity usually requires a fine balance. This is in fact limiting, as the amphiphilic balance has to be optimized for each new polymeric system and is hard to predict.

Antimicrobial activity is usually evaluated using growth inhibition of bacteria or fungi, by reporting the minimal inhibitory concentration (MIC_50_ (50% growth reduction)). On the other hand, toxicity against mammalian cells is predominantly assessed via hemolysis. Although a plethora of different values can be found in literature, the HC_10_, referring to the lowest concentration causing at least 10% of hemolysis is advisable to use. Ideally, this is complemented by cell viability tests using other cell types (e.g., fibroblasts), as results can drastically vary from hemocompatibility. Here the IC_50_ indicating polymer concentration causing 50% of viability loss is commonly reported. To condense the performance of APs into a single number, the selectivity (HC_10_/MIC_50_) was introduced essentially stating how much more active a compound is against bacteria, compared to red blood cells. A similar value often referred to as therapeutic index (IC_50_/MIC_50_) can be constructed to display selectivity over other mammalian cells. Excellent APs will show high values (>100) for both.

Many studies have investigated the suitability of different cationic units and since HDPs were the initial blueprint, mostly nitrogen‐based functionalities were probed. Primary amino groups were found to be superior in many cases,^[^ ^21^, ^22^ ^]^ in particular compared to quaternary ammonium species that feature a permanent charge. A second important motive is the arginine‐inspired guanidinium function. Its peculiar structure leads to a second mode of action based on membrane translocation and precipitation of intracellular components.^[^ ^16^ ^]^ In particular the interaction with genetic material can lead to selective action,^10^ as in contrast to bacteria, in mammalian cells DNA is protected by the nucleus. It should be noted that such a mechanism has also been reported for amino‐based polymer.^[^ ^11^ ^]^ Moreover, bacteria within mammalian cells can be addressed due to the membrane diffusability of guanidine‐based APs.^[^ ^23^ ^]^

A further parameter is the length of the polymer. As most HDPs possess less than 50 amino acids, polymers were usually designed to be in the same molar mass range. Indeed, some studies investigating this parameter find that while antimicrobial activity is not affected by the polymer length, hemotoxicity increases with molar mass, leading to reduced selectivities.^[^ ^18^ ^]^ In other examples, also antibacterial activity scales with molar mass.^[^ ^24^ ^]^ This effect is also dependent on the type of bacteria targeted as in gram‐positive strains, larger polymers can be prevented from reaching the membrane by a sieving effect of the thick cell wall (cell envelope displayed in Figure 1b).^[^ ^25^ ^]^ These findings are however only based on linear copolymers. As will be demonstrated later, structurally confined APs featuring, e.g., a bottle brush architecture can be active despite high molar masses.^[^ ^26^ ^]^ Moreover, a switch from amine to guanidine units can reverse the observed trend.^[^ ^27^ ^]^ A further aspect is the dispersity of APs. Although higher dispersity has been associated with an increase in hemotoxicity,^[^ ^18^ ^]^ a systematic investigation of this matter is still missing.

The polymer backbone also impacts the bioactivity of APs. However, it is challenging to deconvolute the influence of different parameters like flexibility (and the resulting spatial arrangement), polarity (and its influence on the amphiphilic balance), or functional substructures leading to specific interactions (e.g., via hydrogen bonding). Poly(norbornene),^[^ ^17^ ^]^ nylon,^[^ ^19^ ^]^ or poly(peptidic) structures^[^ ^19^ ^]^ belong to the stiffer backbones used, enabling preorientation of subunits. Poly(carbonates),^[^ ^16^ ^]^ poly(oxazoline)s,^[^ ^11^ ^]^ as well as polymers based on acrylates/acrylamides,^[^ ^28^ ^]^ or methacrylates^[^ ^29^ ^]^ are also reported frequently (Figure 1a).

The precise interaction mechanism of APs with the membrane is still not fully understood. Although HDPs can form defined structural units like toroidal pores or barrel staves,^[^ ^28^ ^]^ this is unlikely to happen for nonsequence defined polymers. APs are often described to permeabilize biological membranes, which can be investigated using liposomal membrane models (giant unilamellar vesicles, GUVs) in dye leakage experiments.^[^ ^17^ ^]^ But also the direct depolarization on bacterial cell membranes can be probed in vitro.^[^ ^29^ ^]^ As mentioned before, there are other examples where intracellular interactions seem to play the main role in the antibacterial activity. This can for instance be visualized by electron microscopy studies. Although this is predominantly linked to guanidinium function,^[^ ^10^, ^16^ ^]^ ammonium‐based polymers have also been reported to follow this mechanism of action.^[^ ^11^ ^]^

Similar to intrinsically disordered HDPs, APs with a random sequence of subunits can also adopt a facial amphiphilicity when in contact with the membrane.^[^ ^15^ ^]^ It was also shown that APs can interact preferentially with one type of lipids within a membrane and reorganize its structure.^[^ ^30^ ^]^ This also leads to the hypothesis that APs can catalyze their own binding by recruiting more APs from solution once bound to the interface. Zooming out slightly, APs have been shown to alter membrane morphology, as demonstrated, e.g., by electron microscopy studies revealing formation of protrusions and blebs on the surface of AP‐treated bacteria.^[^ ^24^ ^]^ The overall mechanism is highly sensitive to polymer properties as subtle changes in chemical identity of hydrophobic groups, can lead to a change between burst of GUVs versus pore formation.^[^ ^31^ ^]^ Still, membrane models are highly simplified and bear potential for misinterpretation, for instance due to fusion of GUVs.^[^ ^32^ ^]^

After this general overview we want to focus on certain aspects in AP‐based research that have gained importance in the last years. It should be emphasized that this is not an exhaustive collection of studies, and for a broader picture the reader is referred to other reviews of the field.^[^ ^33^, ^34^ ^]^ In our review, the terms antimicrobial or antibacterial polymers exclusively refer to soluble cationic polymers that are in some way inspired by HDPs. This specifically excludes any bulk materials or surface coating in the context of antifouling. Moreover, we focus strongly on bacteria, while equally important antifungal activity is only mentioned for selected examples.

## 3 Polymer Architecture

**Figure 2:**
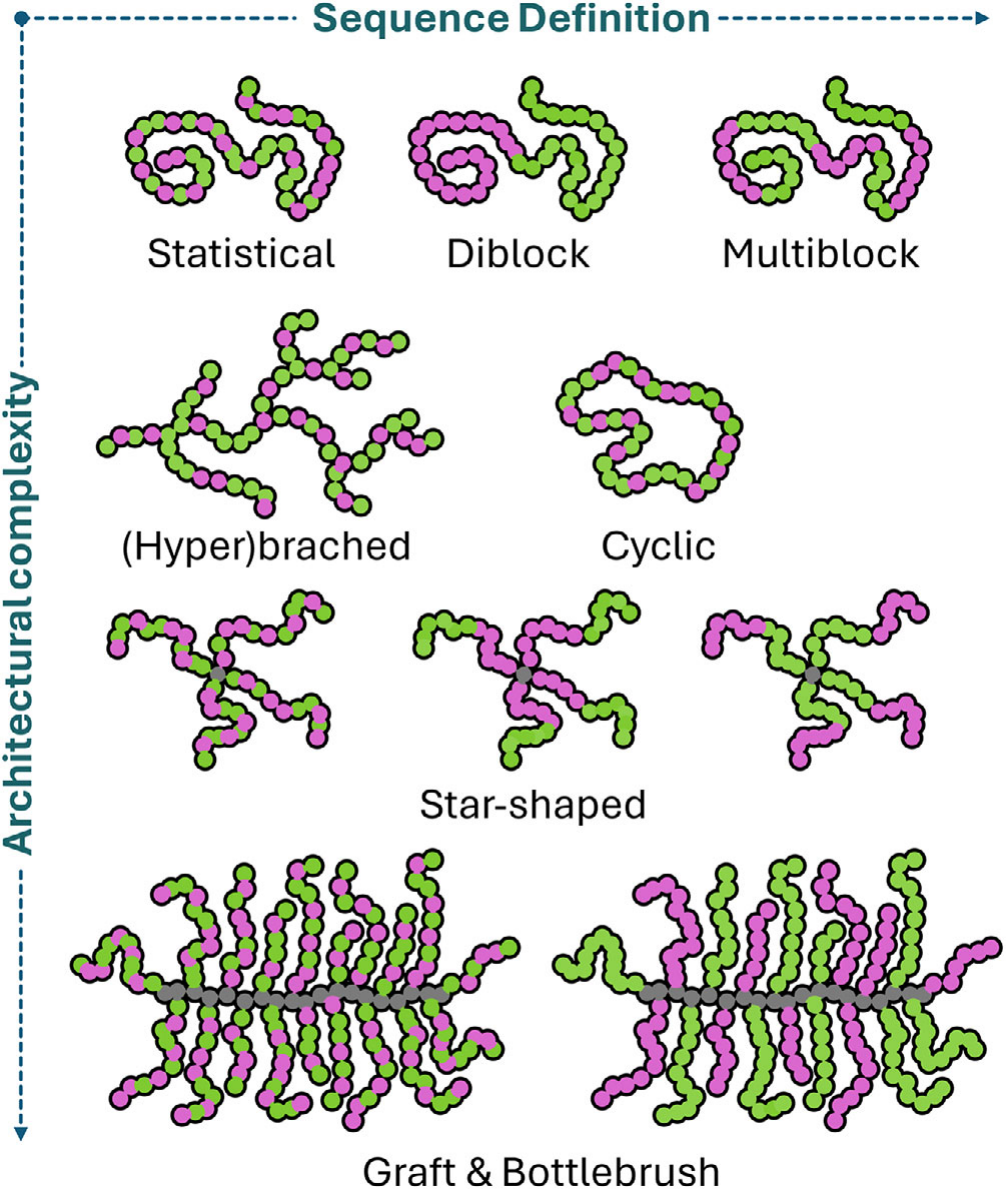
Illustration of structural complexity in APs.

An aspect that was only investigated more closely in the past decade is the architecture or topology of APs. Next to linear copolymers that most closely mimic the primary structure of HDPs, there are other possibilities. Using grafting strategies, star, graft, or bottle brush copolymers can be constructed depending on the nature of the core motif (Figure 2). Also, branched and cyclic APs are described either increasing or decreasing the number of end groups in a single macromolecule.

One important aspect is the localization of functional subunits within the polymer. Although a perfect control of sequence like in peptides is usually not achievable, segregation of monomers in distinct blocks can influence bioactivity. One example is the comparison of diblock and statistical copolymers of equal composition, where hemolysis was reduced for diblocks, likely due to unimolecular self‐assembly.^[^ ^35^ ^]^ Further segregation into multiblock copolymers also leads to distinct differences in bioactivity.^[^ ^36^ ^]^ Here, separation of hydrophobic building blocks led to micelle formation which also diminished antimicrobial activity. This is in line with a second study where polymers lost antimicrobial activity when hydrophobic and hydrophilic segments were separated.^[^ ^37^ ^]^ However, when overall amphiphilicity is reduced (omitting self‐assembly), monomer separation in multiblock copolymers can lead to a drastic increase in selectivity associated to reduced hemotoxicity.^[^ ^8^ ^]^ This was linked to altered physicochemical properties of the respective polymers as a result of monomer segregation.

AP performance can also be modulated by changing polymer architecture. A hyperbranched topology could for instance increase biocompatibility, as hemotoxicity was reduced 4‐fold compared to linear analogues.^[^ ^37^ ^]^ Although the number of end groups is increased in hyperbranched APs, it can also be decreased by using a cyclic topology. In a detailed comparison Fu, Boyer, and coworkers compared APs that were cyclized by a hetero‐Diels–Alder reaction with their linear counterparts.^[^ ^38^ ^]^ Cyclic structures showed generally weaker antimicrobial activity which was attributed to limited conformational freedom of the chains. However, this also led to reduced toxicity against mammalian cells leading to improved selectivity.

Also a star‐shaped topology can be beneficial as shown for poly(peptides) grafted from dendritic core structures.^[^ ^19^ ^]^ However, while the overall performance is impressive, increase of antibacterial activity is only 2‐ fold when mass‐based units are considered instead of molar units (which are not helpful because of the large difference in molar mass between linear and star‐shaped copolymers). Still, decreased flexibility of AP arms in star copolymers can reduce toxicity against mammalian cells.^[^ ^39^ ^]^

However, when monomer units are segregated in star‐shaped copolymers by introduction of blocky arms, antimicrobial activity can be increased 4‐fold along with reduced hemotoxicity.^[^ ^40^ ^]^ Optimal results were achieved only when the cationic segments were placed closer to the core of the structure, highlighting the impact of sequence control.

Graft copolymers and molecular bottle brushes (BB)s are another fascinating architecture in this context. Hedrick and Yang demonstrated that graft‐copoly(carbonate)s exhibit improved activity and selectivity compared to linear copolymers.^[^ ^41^ ^]^ This was also shown for a cellulose backbone, where degradation of the structure to linear polymers reduced activity strongly.^[^ ^42^ ^]^ For copolymers with reduced amphiphilicity, our group could show a drastic increase in bioactivity and selectivity for BB structures compared to linear analogues.^[^ ^43^ ^]^ It was also shown that BB copolymers do not cluster on the membrane interface but penetrate much further into the lipid domain of the membrane compared to linear counterpart, in part explaining their increased activity.^[^ ^44^ ^]^ Moreover, the aspect ratio of BB‐APs was found to be essential for their bioactivity. For equal composition, high aspect ratio BBs (long backbone, short grafts) were much less active and selective when compared to low aspect ratio BBs (short backbone, long side chains).^[^ ^26^ ^]^ A likely reason for this effect is intramolecular self‐assembly resulting in core‐shell structures that unfold upon membrane contact. A similar effect was also reported for linear polymers where phase segregation led to the formation of single‐chain nanoparticles.^[^ ^45^ ^]^ In line with previous findings on diblock copolymers,^[^ ^37^ ^]^ also for BBs with hetero‐grafts (containing cationic or hydrophobic units), self‐assembly led to a loss of antimicrobial activity.^[^ ^29^ ^]^

Overall, both sequence and architecture of APs are essential parameters as they alter properties (e.g., amphiphilicity) and enable multivalent binding. Self‐assembly has to be considered in this context, as intramolecular association can improve bioactivity, while intermolecular micellization can diminish activity by masking hydrophobic qualities. In general, chain mobility seems to strongly influence bioactivity.

## 4 Stimuli‐Responsive Antimicrobial Polymer Systems

**Figure 3:**
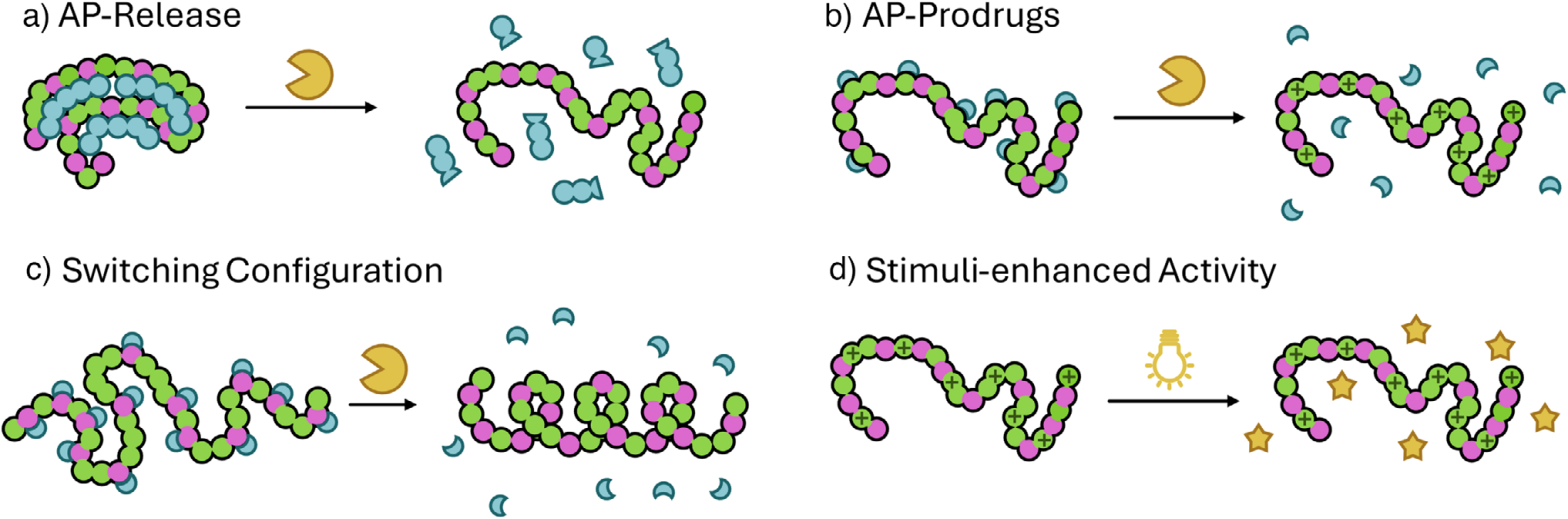
Schematic illustration of different modes of stimuli‐responsive APs. a) AP is released from, e.g., polyplexes, b) functional subunits (e.g., cationic charges) are deprotected upon stimulation, c) stimulation switches AP configuration and thus activity, d) stimuli induces an additional (synergistic) mode of action.

Although the selectivity of APs is mostly determined by structural fe, specific targeting of certain pathogens, using smart or stimuli responsive materials, would further improve applicability. Herein, systems including stimuli‐responsive APs, offering control over the release or activation of the desired therapeutic effect are reviewed. We defined a categorization based on the mechanism of action of the respective AP construct (Figure 3).

The first category already contains the final AP, which is however shielded, covalently or physically with another component, rendering the materials dormant until release (Figure 3a). This approach is used to reduce undesired interaction of the AP with healthy cells. Fernandez‐Trillo et al. synthesized poly interelectrolyte complexes (PIC) containing antimicrobial branched poly(ethylene imine) (PEI) and anionic peptides cleavable by elastase B (LasB), which is produced by *P. aeruginosa*.^[^ ^46^ ^]^ To investigate the antibacterial activity they compared two different bacteria strains, one secreting and one lacking LasB, (PAO1V vs. ΔLasAB). After an incubation period, required for the enzyme to secrete, the PICs incubated with PAO1V reached up to 25% of PEI´s antimicrobial activity, while activity for ΔLasAB stayed constantly low. Palermo et al. used self‐immolative polymers to mitigate hemolytic activity.^[^ ^47^ ^]^ A poly(benzylether) was employed as main chain, and PEG chains and ammonium groups were attached to this backbone as pendants. Using chemically triggered depolymerization, induced by fluoride ions, the main chain was depolymerized, decreasing hemotoxicity while ammonium subunits still exerted antibacterial activity. As such, a chemically triggered increase in selectivity was achieved. Regarding structures featuring a degradable backbone, a stimulus can also be applied to decrease the antibacterial activity upon depolymerization, mitigating the environmental footprint. For APs consisting of a cellulose backbone grafted with linear AP chains, degradation by naturally occurring cellulase decreased the antibacterial activity of the components by an approximate factor of 8 against *E. coli*, or 2.4 against *S. aureus*.^[^ ^42^ ^]^

The second category contains AP prodrugs, possessing protected active sites (mostly positive charges), that are unmasked via a stimulus to toggle bioactivity (Figure 3b). Wong et al. reported an UV‐light responsive polymer, P_ONB,_ where primary amine groups were masked with an ortho‐nitrobenzyl protection group.^[^ ^48^ ^]^ Light irradiation (300 nm) for 8 h cleaves these groups and converts P_ONB_ to P_Photo_. The efficiency of the concept was investigated by comparing MIC values, which showed a decrease from >120 µg mL^−1^ for P_ONB_, to 30 µg mL^−1^ for P_Photo_, while the MIC of the polymer before protection was 16 µg mL^−1^. Although UV‐light might not be suitable for downstream applications, the study shows good proof of concept for light responsive APs.

Later, the same group reported a smart galactosidase‐responsive antimicrobial dendron with capped cationic group that could be activated via β‐galactosidase to uncage primary amines.^[^ ^49^ ^]^ This resulted in an ∼4‐fold increase of activity against different bacteria strains but also an increase in hemotoxicity.

Another class are APs that switch configuration upon stimulation. Here, activity is dependent on specific configuration of the polymer, which is altered upon, e.g., removal of a part of the system, resulting in increased activity (Figure 3c). In 2017 Yin and Cheng *et al.* studied the effect of phosphatase, found in bacteria‐infected tissue, on a poly(peptide) made of *N*‐carboxy anhydride derivatives of l‐glutamate and l‐tyrosine which forms a helical structure according to circular dichroism spectra.^[^ ^50^ ^]^ Phosphorylation of tyrosine residues converts this polymer into a random coil structure, diminishing the activity against *S. aureus* 8‐fold. In the presence of a phosphatase, the helical form and its activity was restored. Hedrick and Yang et al. synthesized a library of random and block copolymers, containing positively charged guanidinium groups and negatively charged acid groups.^[^ ^51^ ^]^ At physiological pH (7.4), polymers form inactive coacervate complexes, while in a more acidic environment (pH 3) protonation results in formation of antibacterial micelles. In particular, block copolymers showed a distinctly increased activity at lower pH values with bacterial survival rates below 10% (as compared to around 90% at physiological pH) against *Helicobacter pylori*.

The last category comprises APs, which increase their activity in a stimuli responsive manner (Figure 3d). This is achieved by activation of a second mode of action which works synergistically with the main mechanism of the AP. Systems including inherently active synergistic components, requiring no external stimuli, are addressed in the next chapter. Palermo and coworkers synthesized APs comprising an oligo(thiophene) backbone featuring positively charged pendants which renders the structure antimicrobially active.^[^ ^52^ ^]^ Additionally, due to the aromatic backbone, such polymers can absorb visible light and react with oxygen to produce reactive oxygen species (ROS), featuring antibacterial activity and leading to a 10‐fold increase upon irradiation (MIC_dark _= 4 µg ml^−1^ vs. MIC_light _= 0.046 µg ml^−1^ against *E. coli*). This strategy was also applied by Boyer and colleagues synthesizing copolymers containing a zinc tetraphenyl porphyrin monomer (ZnTPP‐Ac), which absorbs visible light and generates ROS.^[^ ^53^ ^]^ Depending on composition, a 2‐ to 8‐fold increase in activity against *S. aureus* and *P. aeruginosa* was observed when exposed to green light. Interestingly, ZnTPP‐Ac also acted as an intrinsic catalyst in the PET‐RAFT polymerization that produced the APs. Li et al. designed self‐assembled core‐shell structures with black phosphorus quantum dots as near‐infrared‐(NIR)sensitizer in the core peripherally coated by *S*‐nitrosocysteamine as nitric oxide (NO) donor and cationic amphiphilic copolymers as the shell.^[^ ^54^ ^]^ NIR light led to the production of NO and increased antibacterial efficacy.

Stimuli‐responsive APs offer elevated control over bactericidal activity, increasing efficacy and are able to minimize the hemolytic effects. Diverse triggers can be used as the stimuli, namely enzymes, light, pH, and depolymerization based on the purpose to evoke the activity or suppress AP functions. Such stimuli‐responsive APs are inspiring tools to combat resistant pathogens with reduced side effects and potential spatial‐temporal control of activity.

## 5 Synergistic Strategies Including APs

**Figure 4:**
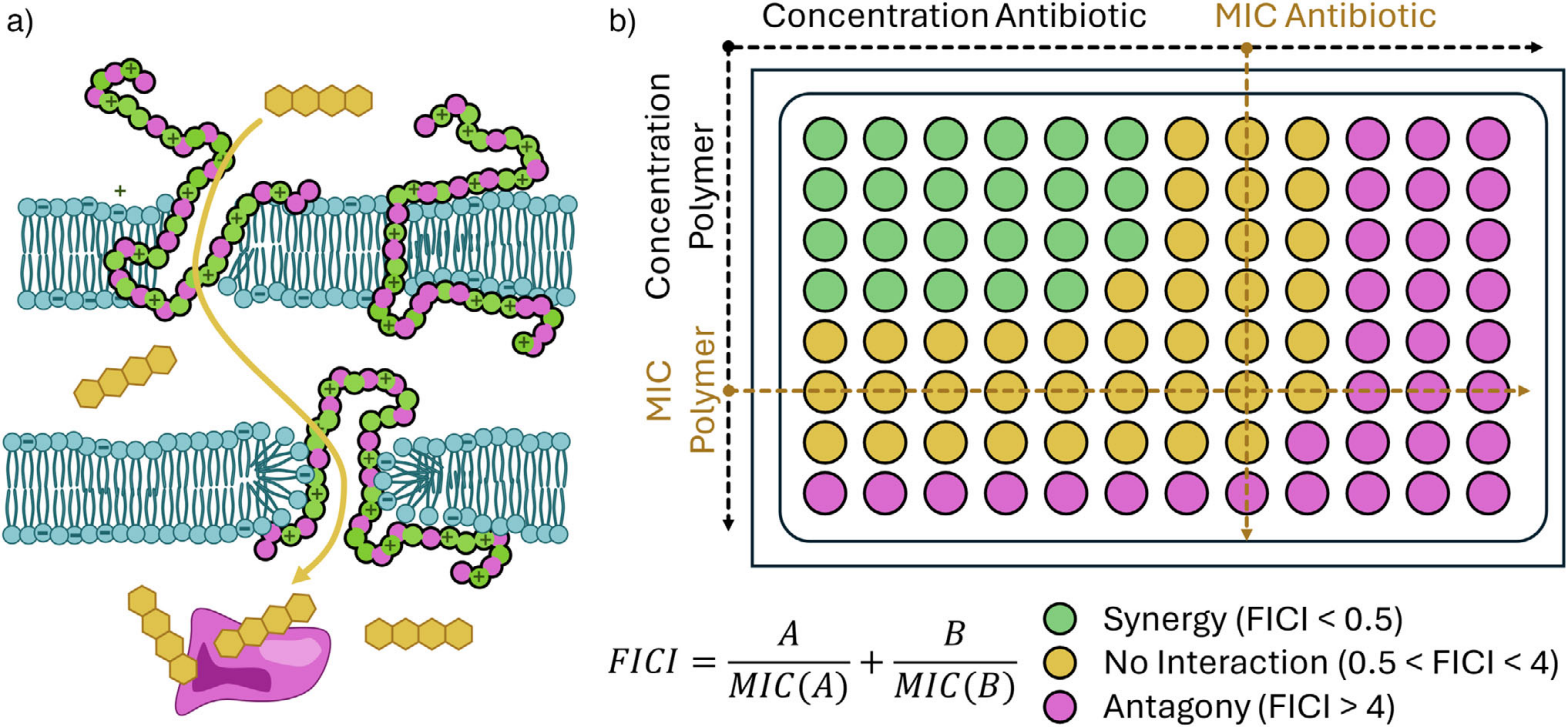
a) Schematic representation of synergistic interaction between an antibiotic (yellow) and an AP leading to increased membrane translocation of the antibiotic. b) Schematic example of a checkerboard assay in a 96 well‐plate format with a log 2 increase in concentration of both polymer and antibiotic. Inhibition in the green wells indicates synergy, inhibition up to the yellow area indicates no interaction and inhibition (only) in the pink region represents an antagonistic relationship between the drugs.

Within this chapter we want to highlight therapeutic approaches, where an AP is combined with a second antimicrobial drug or mechanism to achieve a synergistic effect. This is often probed by a checkerboard assay, where both substances are applied in a matrix of varying concentrations (Figure 4b). The fractional inhibitory concentration index (FICI) is used as benchmark and a FICI < 0.5 indicates synergistic interaction, where the combination is more active than the sum of the individual drugs.

One potential synergy arises when membrane active APs are combined with antibiotics that have an intracellular target. Such concepts are already used employing HDPs as the membrane‐lytic component.^[^ ^55^ ^]^

This effect was demonstrated for APs using cationic poly(carbonates) functionalized with Vitamin E, that were coadministered with the antibiotic colistin leading to FICIs as low as 0.11 for *P. aeruginosa*. The effect was ascribed to the membrane depolarization of the AP enabling uptake of the antibiotic (as visualized in Figure 4a).^[^ ^56^ ^]^ Similarly, APs based on acrylates and acrylamides can be utilized to enhance the activity of doxycycline and colistin.^[^ ^57^ ^]^ Against *P. aeruginosa*, an FICI of 0.38 were achieved corresponding to a 4‐fold increase in activity. In another report poly(aspartate)‐derived APs (carrying quaternary ammonium groups) were combined with Rifampicin, which inhibits RNA polymerases. In optimal combinations an 8‐fold increase in activity (FICI = 0.1875) could be detected against *P. aeruginosa*, a gram‐negative pathogen which is comparably insusceptible to this type of antibiotic.^[^ ^58^ ^]^ Interestingly, the combination with cationic polymers and antibiotics can also be used to restore sensitivity of resistant strains to conventional antibiotics (methicillin‐resistant *S. aureus* (MRSA) to oxacillin, *Enterococcus* f*aecalis* to vancomycin, *E. coli* to streptomycin).^[^ ^59^ ^]^ The same polymer combining chitosan and oligo(lysins) also acted synergistically in the treatment of biofilms in vivo. A similar system was also reported to actively disable bacterial resistance mechanisms.^[^ ^60^ ^]^ Also, in the context of antifungal treatment, synergies of acrylamide‐based APs with antimycotics (fluconazole, caspofungin), with FICI's around 0.3 have been reported, while amphotericin B showed no interaction.^[^ ^61^ ^]^

Also the combination of the essential oils Carvacrol or Eugenol with APs resulted in synergistic interaction as expressed by an improved biofilm eradication of both substances in combination.^[^ ^62^ ^]^ It should be noted that in this case also the polymer topology was essential as only block copolymers were able to encapsulate the oils in a micellar fashion and thus deliver it into biofilms. Similar to APs producing NO upon irradiation,^[^ ^54^ ^]^ it was shown that APs containing primary amines can bind NO and deliver it into the biofilm.^[^ ^63^ ^]^ As only the polymer with NO bound to the amine function, and not the simple mixture of the components shows synergy, it is likely that here the polymer acts as delivery agent in addition to its membrane activity. Also, the combination of Ag nanoparticles decorated with antimicrobial polymers show improvements upon combination. The study suggests that membrane damage enables Ag^+^ ions to enter cytoplasmic space, causing damage by ROS generation.^[^ ^64^ ^]^

Such synergistic effects can also be achieved by combining APs with different modes of action. Hedrick and Yang described poly(carbonate)s carrying either guanidinium functions or quaternary amines, which both possess antimicrobial activity. The combination of both showed synergistic interaction against *P. aeruginosa* (FICI = 0.31) and multidrug resistant *Acinetobacter baumannii* (FICI = 0.25), an effect that was ascribed to membrane permeabilization of ammonium‐based APs, enabling translocation of guanidinium‐based APs to the cytosol where they precipitate intracellular components.^[^ ^65^ ^]^

Concluding this chapter, there is tremendous potential in the co‐administration of APs and other antibiotics in particular as resistant strains can be re‐sensitized for the treatment with conventional antibiotics. This in fact might be a highly promising way for future applications of AP in clinics by supplementing antibiotic treatment instead of replacing it.

## 6 Toward Clinical Applications

For a transfer of APs into clinics, clinical studies need to be conducted. The first step in this direction are in vivo tests. However, up to now only a limited number of examples have been tested in such settings, a selection of which is presented here.

The most straight forward way of applying APs is the treatment of open wounds to prevent biofilm formation. This has been demonstrated on an infection model in rats using MRSA.^[^ ^66^ ^]^ The poly(sulfonium) AP achieved similar results as the positive control (Vancomycin). Also, an AP featuring a guanidine unit in the backbone helped to prolong the lifetime of mice with an infected wound.^[^ ^10^ ^]^

Ammonium‐based poly(peptoid) APs showed increased efficiency in biofilm reduction (related to MRSA) compared to Vancomycin, without any obvious adverse effects on the organism of mice.^[^ ^67^ ^]^ Another pseudo‐peptidic AP used in this context is a poly(oxazoline)‐based polymer with primary ammonium groups. Here MRSA count in a rat wound model could be reduced to a similar extend as with Vancomycin.^[^ ^11^ ^]^

It is worth noting that invertebrates can be a useful alternative to small mammals due to similarities in the immune system. A study by Perrier et al. using a family of guanidinium and ammonium copolymers was tested in *Galleria mellonella* infected with *S. aureus* and showed no toxicity against the animal, as well as improved survival rates.^[^ ^68^ ^]^

A study on mice using guanidinium containing poly(carbonate)s found an LD_50_ of 44 mg kg^−1^ as well as no obvious immune response.^[^ ^16^ ^]^ Pharmacokinetic revealed a half‐life around 17 min, which is comparable to that of carbapenem antibiotics. Noticeably, the biodistribution indicates penetration into the blood stream even after intraperitoneal injection. Multiple resistant infections were successfully treated in a mouse model with a wide therapeutic window. Impressively, the polymers could also efficiently treat an infection with fecal matter, containing both gram‐negative and gram‐positive bacteria, thus displaying one of the most noteworthy advantages of APs: broad spectrum activity. A similar AP was used to successfully treat *K. pneumoniae* lung infections in mouse models.^[^ ^69^ ^]^

Guanidinium‐based poly(oxazoline)s were used, both in topical and by intravenous application to treat fungal infection (*Candida albicans*). They showed a 20 min half‐life and treatments were similarly successful as established antifungal drugs without any obvious damage to organs.^[^ ^70^ ^]^ For similar APs with a shorter alkyl spacer, it was shown that the polymer could cross the blood brain barrier and treat a fungal meningitis in mice.^[^ ^71^ ^]^

Although ultimately it would be ideal to develop APs with oral availability as some common antibiotics possess, the studies that were conducted in the last years are highly promising steps toward a future application in humans.

## 7 Summary and Outlook

Membrane active antimicrobial polymers are a promising class of materials in the war against AMR. In the past decades, fascinating insights about structure–property relationships and mechanisms‐of‐action have been generated. Still there is much more to understand, and compared to HDPs, the polydisperse nature of APs makes this much more difficult.

However, regarding future clinical applications of APs, it is worth noting that due to their polydisperse nature, nonpeptidic structure, and unspecific targets, the development of resistance against them is highly unlikely, as has been demonstrated for multiple systems. In this context it can also be noted that this aspect mitigates one of the largest drawbacks of newly developed antibiotics. If no resistance is expected, APs would not have to be used as last‐resort drug creating a larger financial incentive for the pharma industry. Their non‐uniformity is of course also a disadvantage regarding approval processes.

A major challenge in AP development is the need to individually optimize factors such as amphiphilic balance and topology for each new system. Elucidating and understanding the impact of such parameters detached from each other might enable a more generalized recipe for APs. In this context machine learning tools could prove incredibly useful in the future, with first reports already showing promising results.^[^ ^72^, ^73^ ^]^

Also, the specific interaction with the membrane has to be understood better to enable rational design of APs.

On the applications side, more in vivo studies would be required to press for a future clinical application. More studies including pharmacokinetics and long‐term exposure would give more insight into the interaction of APs with the body.

Ultimately, it would be terrific if APs could be applied like conventional antibiotics. However, due to their inherent cationic nature, they are perhaps too interactive for this to be achieved without the aid of drug delivery systems. As such, stimuli‐responsive systems containing AP prodrugs could be a further way to a systemic application, while some studies already show decent pharmacokinetics of bare APs. However, instead of shining alone, APs might be best suited to work in synergy with antibiotics. Resistance development could not only be reduced, but even already resistant strains could also be resensitized to antibiotics. As polymers that performed well in vivo were to a large extent guanidinium‐based, this building block could be most promising for further developments.

The future will tell how AMR will develop, but it is likely that current measures to combat resistance development will not lead to a holistic solution. As such APs could be an important weapon within the arsenal against multidrug resistant bacteria and fungi.

## Conflict of Interests

The authors declare no conflict of interest.

## Data Availability

Data sharing is not applicable to this article as no new data were created or analyzed in this study.
